# Association between *AXIN1* gene polymorphism (rs9921222) of WNT signaling pathway and susceptibility to osteoporosis in Egyptian patients: a case-control study

**DOI:** 10.1186/s12891-023-06644-y

**Published:** 2023-06-28

**Authors:** Eman Saad Nassar, Rehab Elnemr, Ahmed Shaaban, Asmaa Abd Elhameed, Raghda Saad Zaghloul Taleb

**Affiliations:** 1 Department of Clinical and Chemical Pathology, Faculty of Medicine, Alexandria University, Alexandria, Egypt; 2 Department of Physical Medicine, Rheumatology and Rehabilitation, Faculty of Medicine, Alexandria University, Alexandria, Egypt; 3 Department of Internal medicine, Rheumatology & Immunology division, Faculty of Medicine, Alexandria University, Alexandria, Egypt; 4 Biomedical Informatics and Medical Statistics Department, Medical Research Institute, Alexandria University, Alexandria, Egypt

**Keywords:** Osteoporosis, WNT pathway, *AXINI*, Gene polymorphism, Egyptian population

## Abstract

**Background:**

Osteoporosis (OP) is the most prevalent metabolic bone disease. Numerous genetic loci are strongly related to OP. *AXIN1* is a significant gene that serves an important role in the WNT signaling pathway. The aim of this study was to explore the association between the *AXIN1* genetic polymorphism (rs9921222) and OP susceptibility.

**Methods:**

A total of 101 subjects were enrolled in the study (50 patients with OP and 51 healthy individuals). Genomic DNA was extracted from whole blood using the QIAamp DNA Blood Mini Kit, and the *AXIN1* gene polymorphism (rs9921222) was genotyped by TaqMan allelic discrimination assays. A logistic regression analysis was used to assess the association between genotypes and OP risk.

**Results:**

We found that *AXIN1* rs9921222 had a significant association with the susceptibility of OP under the homozygote model (TT vs. CC: OR = 16.6, CI = 2.03–136.4, *p* = 0.009), (CT vs. CC: OR = 6.3, CI = 1.23–31.8, *p* = 0.027), recessive genetic model (TT vs.TC-CC: OR = 13.6, CI = 1.7–110.4, *p* = 0.015), and the dominant model (TT-TC vs. CC: OR = 9.7, CI = 2.6–36.3, *p* < 0.001). Allele T was significantly associated with OP risk (T vs. C: OR = 10.5, CI = 3.5-31.15, *p* = 0.001). There was a statistically significant difference between genotypes in mean platelet volume (*p* = 0.004), and platelet distribution width (*p* = 0.025). In addition, lumbar spine bone density, and femur neck bone density were significantly different between genotypes (*p* < 0.001).

**Conclusion:**

*AXIN1* rs9921222 was associated with OP susceptibility in the Egyptian population and should be considered a potential determinant risk for OP.

## Background

The prevalent systemic skeletal illness, osteoporosis (OP), known as the “invisible killer,“ is characterized by decreased bone mass and altered bone microarchitecture, which ultimately cause skeletal fragility and fractures in many sites [1, 2]. It’s considered the most prevalent metabolic bone condition that affects more than 200 million people worldwide.

Bone mineral density (BMD) is a crucial indicator for the diagnosis of OP as well as a highly significant risk factor for fracture [3]. It has a heritability of 50–80% [4, 5], which is very high.

According to numerous research findings [6, 7], it is believed that genetic variables, specifically gene polymorphism or gene mutation, play a significant role in the complex pathogenic process of primary OP. Numerous genetic loci are strongly related to BMD, according to genome-wide association studies (GWAS) [8–10].

The Wnt signaling system is essential for modulating bone growth, bone mass, and osteoblast differentiation [11]. The activation of β-catenin by Wnt ligands is required for osteoblast differentiation. The Wnt/β-catenin signaling pathway, can direct the development of bone marrow mesenchymal stem cells [12]. In OP patients, there are less common bone marrow mesenchymal stem cells, fewer bone-differentiating cells, and fewer osteoblasts are produced [13, 14].

Axis inhibition protein 1 (*AXIN1*) is a significant gene that is located on chromosome 16p13.3 [15]. *AXIN1* was reported to be a key suppressor of Wnt signaling pathway and an important scaffold for the turnover of β-catenin [16]. SNP rs9921222 is an intronic variant of *AXIN1* that correlates with BMD. However, just one study has described the biological mechanism of SNP rs9921222 in OP [17]. Moreover, research has demonstrated that polymorphisms in the *AXIN1* gene can be employed as a new genetic signal for bone mineral content [10]. Thus, more studies of the genetic variation of *AXIN1* linked to OP are required which will help to clarify the mechanism of *AXIN1* affecting the occurrence and development of OP.

## Materials and methods

### Study participants

A total of 101 individuals were enrolled in the study (50 patients with primary OP diagnosed according to WHO diagnostic criteria (T-score ≤ -2.5) that was obtained by measuring BMD of the hip and lumbar spine by using dual-energy X-ray absorptiometry (DXA) [18] and 51 apparently healthy age and sex matched controls) from January 2022 until December 2022. Patients with a history of malignancy, or patients with severe liver or kidney disease, any autoimmune disease as rheumatoid arthritis, metabolic or genetic bone diseases including hypothyroidism or hyperthyroidism, Paget disease, osteogenesis imperfecta, and osteomalacia, patients with gastrointestinal diseases as Crohn’s disease were excluded from the study. Additionally, patients who applied steroid hormone or anticonvulsants for 6 months or more and those with previous long-term use (6 months or more) or currently using drugs for OP treatment were also excluded from the study. Bone density scan was used to diagnose OP and to assess the patient’s risk to develop osteoporotic fractures.

#### Sample collection

Eight milliliters of whole blood were collected by venipuncture from antecubital vein under complete aseptic technique into three vacutainer tubes: Four mL blood in BD Vacutainer® red top blood collection tubes containing clot activator (Becton Dickinson and Company, USA) to separate serum that was used for chemistry analysis. Two mL in BD vacutainer® lavender top blood collection tubes containing K_3_EDTA (Becton Dickinson and Company, USA) was used for complete blood count (CBC). Two mL in another BD vacutainer® lavender top blood collection tubes containing K_3_EDTA (Becton Dickinson and Company, USA) was used for molecular analysis to detect *AXIN1* gene polymorphism (rs9921222).

## Laboratory investigations

Serum creatinine, alanine aminotransferase (ALT), calcium, phosphorus, and alkaline phosphatase were measured using chemistry analyzer Dimension RxL Max (Siemens Healthineers, Germany). CBC was performed using ADVIA 2120 (Siemens Healthineers, Germany).

### AXIN1 gene SNP (rs9921222) genotyping

Genomic DNA was extracted from whole blood using QIAamp DNA Blood Mini Kit (Catalog no. 51,104) (QIAGEN, Germany) according to manufacturer’s instructions. DNA concentration and purity were assessed using NanoDrop 2000c Spectrophotometer (ThermoFisher Scientific, USA).

*AXIN1* gene polymorphism (rs9921222) was genotyped by TaqMan allelic discrimination assays (5′ nuclease assay). The PCR reaction was performed in a 20-µL reaction volume containing 20 ng genomic DNA. The PCR reaction mix included 10 µL TaqMan® Genotyping Master Mix (2X) (Applied Biosystems-Life Technologies, USA), 1 µL TaqMan® SNP Genotyping Assay 20x (Assay ID: C__2464929_10, Catalog no. 4,351,379 (Applied Biosystems-Life Technologies, USA)), and 9 µL of DNA sample and DNase-free, RNase-free water. Thermal cycling was performed using Stratagene Mx3000P QPCR system (Agilent, USA) as follows: 95 °C for 10 min for AmpliTaq Gold enzyme activation and 40 cycles of denaturation for 15 s at 95 °C and annealing/extension for 1 min at 60 °C. No template control (NTC) containing nuclease-free water was included in each run as a negative control. The fluorescence profile of each well was detected at the end of each cycle and a graphic presentation of the fluorescence against the number of cycles was plotted. Data processing was performed using Stratagene Mx3000P™ software (MX.PRO software).

Bone density measurement:

BMD was measured by using DXA scan which is the most widely used test to determine bone density. WHO classifies T-score as follows: above − 1 SD is normal, between − 1 and − 2.5 SD is defined as osteopenia, while T-score at or below − 2.5 SD is considered osteoporosis [18].

***Sample size and power calculation*** [19].

Using PASS 2000 software (Power Analysis and Sample Size software (, our sample size of 101 (50 patients and 51 healthy controls) achieved 80% statistical power to detect an odds ratio of at least (OR = 4), for any genotype, at a level of significance 5%, using binary logistic regression analysis [19].

### Statistical analysis

Statistical analysis was performed using SPSS software version 28.0. (Armonk, NY: IBM Corp) [20]. The differences in demographic data were described using frequency, percent, while quantitative data were described using mean, standard deviation (SD). Comparing quantitative data between 2 groups was carried out using independent sample t-test, while categorical data were compared using Pearson Chi Square test. Different genotypes were compared using One Way ANOVA test. The logistic regression analysis was used to calculate the odds ratio (OR) and 95% confidence interval (CI) to assess the association between genotypes and OP risk, while adjusting by age and gender. The association between *AXIN1* rs9921222 and the OP susceptibility among participants was evaluated (OR = 1: this factor has no effect on the susceptibility of OP; OR < 1: this factor can reduce the susceptibility of OP; OR > 1: this factor can increase the susceptibility of OP). Hardy–Weinberg equilibrium (HWE) was calculated by Gene-Calc software (computer software) [21]. All statistical analyses in this study are two-sided tests, and *p* < 0.05 was considered statistically significant.

## Results

### Study participants

The patients group included 10 males (20%) and 40 females (80%); their mean age was 54 ± 8 years. The control group included 9 males (17.5%) and 42 females (82.5%); their mean age was 58 ± 6 years.

There was no statistically significant differences between patients and healthy volunteers regarding age, gender and serum calcium level while statistically significant difference was found regarding ALT (*p* < 0.001), alkaline phosphatase (*p* < 0.001), and serum creatinine (*p* = 0.03); their mean level was higher in patients than control group. Moreover, DXA revealed statistically significant lower T-score of lumbar spine and total femur in patients than healthy subjects (*p* < 0.001). Table 1 summarizes demographic, laboratory, and radiological data of studied subjects.

**Table 1:** Characteristics of studied subjects (n = 101)

	Patients	Control	
	Mean (SD)	Mean (SD)	*p*
**Age (years), n (%)**			
**≤ 60**	30 (60%)	40 (78.4%)	0.045
**> 60**	20 (40%)	11 (21.6%)	
**Gender, n (%)**			
**Male**	10 (20%)	9 (17.5%)	0.76
**Female**	40 (80%)	42 (82.5%)	
**Hemoglobin (g/dl)**	12.4 (0.8)	12.3 (1.2)	0.54
**Platelet count (x10** ^**3**^ **/µl)**	264 (72)	259 (74)	0.75
**MPV (fL)**	12.8 (0.0)	9.5 (1.6)	< 0.001*
**PDW (%)**	13 (2)	15 (2)	< 0.001*
**ALT (U/L)**	35 (7)	29 (7)	< 0.001*
**Serum creatinine (mg/dl)**	0.90 (0.17)	0.76 (0.42)	0.03*
**Serum calcium (mg/dl)**	8.96 (0.81)	9.11 (0.35)	0.25
**Ionized calcium (mg/dl)**	4.38 (0.58)	4.34 (0.40)	0.69
**Serum phosphorus (mg/dl)**	3.97 (0.74)	4.31 (0.49)	0.011*
**Alkaline phosphatase (U/L)**	133.44 (32.05)	106.26 (16.58)	< 0.001*
**Lumbar spine AP T-score**	-2.9 (0.6)	-1.0 (0.1)	< 0.001*
**Left femur total T-score**	-2.46 (0.67)	-1.02 (0.14)	< 0.001*

*Statistically significant by independent t- test; ALT: Alanine aminotransferase; AP: Anteroposterior; MPV: Mean platelet volume; PDW: Platelet distribution width

### Hardy-Weinberg equilibrium

The results of Hardy–Weinberg balance test showed that *AXIN1* rs9921222 was consistent with Hardy-Weinberg equilibrium (x^2^ = 0.047, *p* = 0.976). Minor allele frequency in the patients’ group was 0.6 while in the control group was 0.06.

### Association between AXIN1 rs9921222 SNP and susceptibility risk

Table 2 shows logistic regression analysis for association between the *AXIN1* rs9921222 SNP and OP risk adjusted by age and gender. *AXIN1* rs9921222 had a significant association with the susceptibility of OP under the homozygote model (TT vs. CC: OR = 16.6, CI = 2.03–136.4, *p* = 0.009), (CT vs. CC: OR = 6.3, CI = 1.23–31.8, *p* = 0.027), recessive genetic model (TT vs.TC-CC: OR = 13.6, CI = 1.7–110.4, *p* = 0.015), dominant model (TT-TC vs. CC: OR = 9.7, CI = 2.6–36.3, *p* < 0.001). Allele T was significantly associated with OP risk (T vs. C: OR = 10.5, CI = 3.5-31.15, *p* = 0.001).

**Table 2:** Multiple logistic regression analysis of the association between susceptibility of osteoporosis and *AXIN1* rs9921222 SNP in different genetic models

						95% C.I. for OR			95% C.I. for OR		AIC
Model	Allele/Genotype	Patients	Control	*p*	Unadjusted OR	Lower	Upper	Adjusted OR	Lower	Upper	
**Allele**	T	30	4	0.001*	10.5	3.5	31.15				
	C	70	98								
**Genotype**	TT	11	1	0.009*	16.6	2.03	136.4	13.04	1.5	112.95	200
	CT	8	2	0.027*	6.3	1.23	31.8	5.9	0.99	35.3	
	CC	31	48		1						
**Dominant**	CC	31	48		1						
	TT-TC	19	3	< 0.001*	9.7	2.6	36.3	8.5	2.12	34.35	200
**Recessive**	TT	11	1	0.015*	13.6	1.7	110.4	11.2	1.28	94.8	200
	TC-CC	39	50		1						

OR: Odds ratio CI: Confidence interval *p < 0.05 statistically significant AIC: Akaike information criterion

Adjusted OR: Adjusted for age, serum creatinine, ALT, alkaline phosphatase

The multivariate logistic regression analysis was performed to adjust for the statistically significant parameters (age, ALT, alkaline phosphatase, and serum creatinine). There was a slight difference in the OR after adjusting by these factors; homozygote model (TT vs. CC: OR = 13.04, CI = 1.5–112), (CT vs. CC: OR = 5.9, CI = 0.99–35.3 ), recessive genetic model (TT vs.TC-CC: OR = 11.2, CI = 1.2–94.8), dominant model (TT-TC vs. CC: OR = 8.5, CI = 2.1–34.3). Akaike information criteria (AIC) for accuracy of the models including the 4 parameters adjusting for, was 200 (AIC = 200).

### Differences in demographic, laboratory, and radiographic parameters in different genotypes

There was no statistically significant difference in mean age between genotypes TT (58 years), CT (56 years), and CC (56 years) (*p* = 0.62). Additionally, there was no statistically significant difference in mean hemoglobin level between the 3 genotypes TT (12.3 g/dl), CT (12.1 g/dl), and CC (12.3 g/dl) (*p* = 0.79).

There was statistically significant difference between genotypes in mean platelet volume (MPV) (*p* = 0.004), and platelet distribution width (PDW) (*p* = 0.025). In addition, lumbar spine bone density, and femur neck bone density were significantly different between genotypes (*p* < 0.001). Table 3 summarizes the difference in clinical parameters among *AXIN1* rs9921222 genotypes.

**Table 3:** Clinical parameters according to *AXIN1* rs9921222 genotypes

	Genotype						
	TT		CT		CC		
	Mean	SD	Mean	SD	Mean	SD	*P* value
**Age (years)**	58	8	56	7	56	8	0.62
**Hemoglobin (g/dl)**	12.3	0.4	12.1	1.3	12.3	1.1	0.79
**Platelet count (x10** ^**3**^ **/µl)**	263	85	220	49	267	73	0.12
**MPV (fL)**	12.8	0.0	12.0	1.5	10.7	2.1	0.004*
**PDW (%)**	12	2	14	2	14	2	0.025*
**ALT (U/L)**	35	6	32	6	31	8	0.43
**Serum creatinine (mg/dl)**	0.98	0.16	0.82	0.31	0.81	0.351	0.308
**Serum calcium (mg/dl)**	9.18	0.27	8.77	1.02	9.06	0.59	0.27
**Ionized calcium (mg/dl)**	4.42	0.33	4.40	0.44	4.35	0.52	0.90
**Serum phosphorus (mg/dl)**	3.96	0.48	3.89	0.38	4.19	0.69	0.25
**Alkaline phosphatase (U/L)**	138.45	26.14	122.00	29.21	116.92	28.46	0.064
**Lumbar spine AP T-score**	-3.02	0.28	-2.30	0.90	-1.8	1.0	< 0.001*
**Left femur total T-score**	-2.74-	0.28	-2.18	0.90	-1.53	0.80	< 0.001*

*Statistically significant by one way ANOVA test; ALT: Alanine aminotransferase; AP: Anteroposterior; MPV: Mean platelet volume; PDW: Platelet distribution width

## Discussion

Osteoporosis is a multifactorial skeletal disorder characterized by low BMD and destruction of bone microarchitecture, resulting in increased fracture susceptibility [22]. One of the signal pathways known to play a vital role in normal bone metabolism is the WNT signaling pathway. The WNT signaling cascade promotes bone production while suppressing bone resorption, resulting in a balanced bone remodeling process. Recent research has reaffirmed WNT signaling unavoidable role in OP. *AXIN1* encodes a protein that functions as a negative regulator of WNT signaling pathway [23]. Genetic variants are important determinants of normal bone mass variability and predisposing factors for OP [24]. Surprisingly, the impact of WNT signaling pathway-related gene polymorphisms (*AXIN1*) on OP risk in Egyptian population remains unknown. To the best of our knowledge, this is the first study to investigate the role of *AXIN1* gene polymorphism in Egyptian patients with OP.

SNP rs9921222 is an intronic variant of *AXIN1* that is associated with several phenotypes, including BMD [25], height [26] and iron homeostasis [27], highlighting the significance of this SNP in physiological functions. This SNP has been associated with lower BMD in several populations. Nevertheless, the SNP’s biological role has not yet been found. In the present study, we found that *AXIN1* rs9921222 SNP is a risk factor for OP in Egyptian population (OR > 1). In addition, TT genotype was associated with lower lumbar spine and femoral neck bone density compared to CT and CC genotypes. This finding agrees with Cui et al. who concluded that *AXIN1* rs9921222 SNP is significantly associated with susceptibility to OP in Chinese Han population. Moreover, they found that *AXIN1* rs9921222 SNP is associated with lower L4 spine BMD and higher risk of OP in patients with body mass index (BMI) ≤ 24 [28]. Likewise, Suthon et al. demonstrated that TT genotype at rs9921222 is associated with higher expression of *AXIN1* in osteoblasts in comparison to CC genotype and subsequently lower BMD [17]. These results are supported by Estrada et al. who performed a genome-wide meta-analysis and identified 56 loci including 64 SNPs related to BMD and fracture risk. *AXIN1* rs9921222 SNP was one of the newly discovered loci associated with BMD [29]. Styrkarsdottir et al. [30] found a new BMD signal at *AXIN1* locus that was not previously reported by Estrada et al. Importantly, they reported that the new signal was strongly associated with spine BMD and weakly associated with hip BMD and fractures in subjects of European and East-Asian descent. Similarly, Kim et al. reported that *AXIN1* rs9921222 SNP was also associated with low lumbar spine BMD in Korean cohort. Additionally, they reported that *AXIN1* rs9921222 SNP was associated with increased risk of osteoporotic fractures in other Asian cohorts [31]. Medina-Gomez et al., performed a meta-analysis of 30 epidemiological studies of total BMD comprising American, European, and Australian individuals. They demonstrated that *AXIN1* rs9921222 SNP was associated with total body BMD in European cohort only [32]. Overall, these results strengthen the evidence that *AXIN1* rs9921222 SNP is associated with lower BMD and higher osteoporotic fracture risk in different populations.

The biological function of *AXIN1* rs9921222 SNP in bone has not been extensively studied. Therefore, Suthon et al. investigated the molecular mechanism underlying the regulation of AXIN1 protein by rs9921222 SNP in human osteoblasts. They elucidated that rs9921222 SNP regulates *AXIN1* gene expression through binding of transcription factor GATA4. Allele T has higher binding affinity to GATA4 resulting in higher *AXIN1* gene expression and lower level of transcriptionally active β-catenin. Subsequently, low level of β-catenin represses receptor activator of nuclear factor kappa B ligand (RANKL) transcription in osteoblasts ending up with activation of osteoclast maturation and lower BMD [17]. Although this study provided a clue on the reason behind the difference in phenotype, further experiments are needed to emphasize the role of this SNP in regulation of *AXIN1* gene expression.

Osteoporosis is a disease of elderly; however, genetic variants affecting bone mass maintenance and bone loss may start their effect early in life. Given this fact, Warrington et al. studied 63 SNPs known to influence BMD in adults in a group of children and adolescents to investigate whether they have an impact on bone acquisition rate during adolescence. They reported that *AXIN1* rs9921222 SNP is significantly associated with low BMD at age 13; whereas, weakly associated with rate of change in BMD over adolescence [33]. This weak association may indicate that the function of this SNP does not affect the change of BMD over this age. However, a larger study cohort is needed to confirm this finding. In the present study, the patients’ cohort was adults with mean of age 58 ± 6 years; nevertheless, we recommend studying the effect of *AXIN1* rs9921222 SNP on BMD in Egyptian children and adolescents.

Interestingly, we found MPV significantly higher in osteoporotic patients specifically in TT genotype compared to CC and CT genotypes. Despite the fact that MPV is known to have an important role in the development of OP [34], its association with *AXIN1* rs9921222 SNP has not yet been studied. In concordance with our results, Cui et al. reported that MPV and PDW are significantly higher in osteoporotic Chinese patients compared to healthy individuals; however, they did not study the difference in MPV among *AXIN1* rs9921222 genotypes [28]. In contrast to our results, Akbal et al. found both MPV and PDW significantly lower in osteoporotic patients; yet MPV was not correlated with BMD measurements [35]. They attributed this different finding to the exclusion of patients with confounding factors such as cardiovascular diseases, hypertension, and diabetes mellitus. Another important finding from our study was the significantly lower PDW levels in osteoporotic patients. Of note, PDW was lower in TT genotype compared to CC and CT genotypes. Although not yet definite, PDW was suggested as marker of OP. To confirm this hypothesis, Akbal et al. studied the relationship between PDW and bone mineralization and found that PDW was significantly lower in osteoporotic patients and positively correlated with BMD measurements [35]. As far as we know, no studies investigated the relation between *AXIN1* rs9921222 SNP and platelet indices such as MPV and PDW. Therefore, more studies in this context are needed to endorse our findings.

## Conclusion

We suggest that *AXIN1* rs9921222 SNP should be considered as a potential determinant for risk of OP in Egyptian population. However, we believe that for more comprehensive and precise assessment of the relation between *AXIN1* rs9921222 SNP and the risk of OP, large sample size is required, and the synergistic effect of other factors such as other genetic variants, environment, dietary habits, BMI, and exercise should be taken into consideration.

### Limitations

There are limitations to this study. First, the small sample size of the study limited the generalization of the results. Furthermore, the synergistic effect of other factors such as other genetic variants, environment, dietary habits, BMI, and exercise should be taken into consideration.

## Data Availability

All data generated or analyzed during this study are included in this manuscript.

## Abbreviations

OP: Osteoporosis
BMD: Bone mineral density
GWAS: Genome-wide association studies
AXINI 1: Axis inhibition protein 1
SNP: Single nucleotide polymorphism
DXA: Dual energy X ray absorptiometry
CBC: Complete blood count
ALT: Alanine aminotransferase
NTC: No template control
HWE: Hardy–Weinberg equilibrium
MPV: Mean platelet volume
